# The use of low-density EEG for the classification of PPA and MCI

**DOI:** 10.3389/fnhum.2025.1526554

**Published:** 2025-02-07

**Authors:** Panteleimon Chriskos, Kyriaki Neophytou, Christos A. Frantzidis, Jessica Gallegos, Alexandros Afthinos, Chiadi U. Onyike, Argye Hillis, Panagiotis D. Bamidis, Kyrana Tsapkini

**Affiliations:** ^1^Department of Neurology, Johns Hopkins School of Medicine, Baltimore, MD, United States; ^2^Laboratory of Medical Physics and Digital Innovation, Faculty of Health Sciences, School of Medicine, Aristotle University of Thessaloniki, Thessaloniki, Greece; ^3^School of Engineering and Physical Sciences, College of Health and Science, University of Lincoln., Lincoln, United Kingdom; ^4^Cooper Medical School of Rowan University, Camden, NJ, United States; ^5^Department of Psychiatry and Behavioral Sciences, Johns Hopkins School of Medicine, Baltimore, MD, United States; ^6^Department of Cognitive Science, Johns Hopkins University, Baltimore, MD, United States

**Keywords:** primary progressive aphasia, mild cognitive impairment, classification, electroencephalography (EEG), functional connectivity, energy rhythms

## Abstract

**Objective:**

Dissociating Primary Progressive Aphasia (PPA) from Mild Cognitive Impairment (MCI) is an important, yet challenging task. Given the need for low-cost and time-efficient classification, we used low-density electroencephalography (EEG) recordings to automatically classify PPA, MCI and healthy control (HC) individuals. To the best of our knowledge, this is the first attempt to classify individuals from these three populations at the same time.

**Methods:**

We collected three-minute EEG recordings with an 8-channel system from eight MCI, fourteen PPA and eight HC individuals. Utilizing the Relative Wavelet Entropy method, we derived (i) functional connectivity, (ii) graph theory metrics and extracted (iii) various energy rhythms. Features from all three sources were used for classification. The k-Nearest Neighbor and Support Vector Machines classifiers were used.

**Results:**

A 100% individual classification accuracy was achieved in the HC-MCI, HC-PPA, and MCI-PPA comparisons, and a 77.78% accuracy in the HC-MCI-PPA comparison.

**Conclusion:**

We showed for the first time that successful automatic classification between HC, MCI and PPA is possible with short, low-density EEG recordings. Despite methodological limitations of the current study, these results have important implications for clinical practice since they show that fast, low-cost and accurate disease diagnosis of these disorders is possible. Future studies need to establish the generalizability of the current findings with larger sample sizes and the efficient use of this methodology in a clinical setting.

## 1 Introduction

Neurodegenerative disorders are diverse pathologies and clinical phenotypes, and differential diagnosis and classification require considerable expertise from the clinician. For example, the dissociation between Primary Progressive Aphasia (PPA) and Mild Cognitive Impairment (MCI) is often challenging. PPA is an age-related neurodegenerative syndrome, primarily characterized by a gradual deterioration of language functions but other cognitive functions are impaired as well (Mesulam, 1982; Mesulam, 1987). PPA is usually divided into three variants: non-fluent/agrammatic variant PPA (nfvPPA), semantic variant PPA (svPPA) and logopenic variant PPA (lvPPA), although there are also mixed and unclassified cases (Gorno-Tempini et al., 2011). Each variant is associated with distinct regions of brain atrophy, diverse pathologies (primarily frontotemporal lobar degeneration and Alzheimer’s disease), as well as with diverse neuropsychological profiles (Gorno-Tempini et al., 2011). MCI, on the other hand, is a neurodegenerative syndrome characterized by cognitive decline (in memory only or in more than one cognitive functions) that is over and above what is expected given an individual’s age and education level (Gauthier et al., 2006), and high prevalence of Alzheimer’s pathology (Bennett et al., 2005). Given the overlap in symptoms, confident diagnosis of early PPA versus MCI can be difficult (Faroqi-Shah et al., 2020; Rogalski and Mesulam, 2009). However, accurate diagnosis is important for understanding the trajectory of the patient’s disorder and, consequently, for suggesting appropriate treatments. From a clinical perspective, a quick and task-free way of classifying PPA vs. MCI, will be extremely valuable—especially in low resource settings.

Prior work has primarily focused on automatic classifications of healthy control *(HC)* vs. *PPA* individuals, and the three variants of PPA (Álvarez et al., 2019; Bisenius et al., 2017; Díaz-Álvarez et al., 2022; Matias-Guiu et al., 2019, 2022; Moral-Rubio et al., 2021; Spinelli et al., 2017), as well as on the classification of *HC vs. MCI* (Chriskos et al., 2021; Faghfouri et al., 2024; Fraser et al., 2019; Lagun et al., 2011). To the best of our knowledge, only one study has attempted to classify *PPA* vs. *MCI* patients (Bruun et al., 2019). These studies have utilized data recorded from two main sources: neuroimaging and behavior. Neuroimaging sources include Magnetic Resonance Imaging (MRI) (Agosta et al., 2015; Bisenius et al., 2017; Bruun et al., 2019; Kim et al., 2019; Poonam et al., 2021; Spinelli et al., 2017) and Positron Emission Tomography (PET) (Álvarez et al., 2019; Díaz-Álvarez et al., 2022; Matias-Guiu et al., 2018, 2019, 2020; Slegers et al., 2021), while Magnetoencephalography (MEG) has been used to investigate the differences observed between PPA patients and healthy controls in reactions to linguistic stimuli, but without attempting to automatically classify PPA individuals (Kielar et al., 2018, 2019, 2022). Behavioral sources include audio recordings from which speech features are extracted (Cho et al., 2020; Cordella et al., 2017; García et al., 2022; Mahmoud et al., 2021; Matias-Guiu et al., 2018, 2022; Themistocleous et al., 2021; Zimmerer et al., 2020), as well as other cognitive and language data based on, for example, spelling performance (Neophytou et al., 2019), morphological processing (Stockbridge et al., 2021), semantic knowledge and episodic memory (Hoffman et al., 2017).

The classification accuracy varies considerably in the reviewed works, but several studies have reported high accuracy, especially when advanced classification algorithms were used. Classification of *HC* vs. *PPA* is often achieved with extremely high accuracy using methods such as a Convolutional Neural Network (CNN) (Mahmoud et al., 2021) and a Support Vector Machine (SVM) (Bisenius et al., 2017). In several cases (Agosta et al., 2015; Cordella et al., 2017; García et al., 2022), the two classes used are healthy and non-fluent variant PPA (nfvPPA) with Cordella et al. (2017) reporting a perfect score of 100%. Classification of the three PPA variants has also been achieved with high accuracy, 78%, using a deep neural network on speech utterances (Themistocleous et al., 2021). Classification attempts for *HC* vs. *MCI* have achieved high accuracy, 87%, using SVM algorithms (Lagun et al., 2011), and up to 91% using CNN (Chriskos et al., 2021). The one study that has compared *MCI* vs. *PPA* has achieved relatively low accuracy values (59%), but no advanced classification algorithms were used (Bruun et al., 2019).

Classification accuracy is tightly associated with the classification algorithms used, but also with the type of data used. Neuroimaging data usually allows for higher classification accuracy, yet they are inherently high cost and require the availability of appropriately trained medical and support staff. Behavioral data can be used to achieve good classification accuracy, especially if coupled with advanced classification algorithms. However, while audio recordings can be conducted with simple and low-cost equipment, they are highly time-consuming to collect and preprocess, as well as to extract the information relevant for classification.

To the best of our knowledge, only one study has tried to dissociate PPA from MCI, but this was based on MRI data (Bruun et al., 2019). Given the need for low-cost and time-efficient classification of PPA and MCI, in the current study we used low-density (i.e., 8-channel) Electroencephalography (EEG) recordings to classify between PPA, MCI and HC individuals. To achieve this, we used features extracted from three sources. Utilizing the Relative Wavelet Entropy (RWE) method, we derived (i) Functional Connectivity (FC) values across the 8 channels, as well as (ii) graph theory metrics, and also separately extracted (iii) the energy of various rhythms.

In contrast to behavioral tasks, often a time-consuming endeavor that requires several language-specific tests, we used only 3 minutes of resting-state EEG recordings per individual with an 8-channel system to extract all these features, which is quick to set up and task-free. Importantly, unlike neuroimaging techniques, such as MRI and PET, which are expensive and often non-accessible, EEG is a low-cost option for studying the brain and is widely used. Previous research has shown abnormalities in the EEG profiles of PPA (Grieder et al., 2016; Mesulam, 1982; Utianski et al., 2019, 2022) and MCI (Baker et al., 2008; Grunwald et al., 2002; Jelic et al., 2000; van der Hiele et al., 2007) individuals. However, no study has attempted to distinguish PPA from MCI based on their EEG profiles. The current study aimed to address this gap and automatically classify PPA and MCI individuals using features extracted from short EEG recordings.

## 2 Materials and methods

### 2.1 Participants and experimental procedure

The data used in this paper originate from two different datasets. The first one, containing the healthy elderly and the MCI group, was derived from the Long Lasting Memories (LLM) study (Frantzidis et al., 2014). Participants underwent detailed clinical examinations. MCI diagnosis was based on the Petersen criteria (Petersen, 2004). The MCI group comprised of eight right-handed individuals (mean age = 67.8, SD = 4.6) with MMSE scores ranging from 24 to 25 (mean = 24.75, SD = 0.46) and MoCA scores ranging between 22 and 24 (mean = 22.88, SD = 0.83). The MCI individuals were diagnosed as *amnestic* MCI following neuropsychological assessment which was part of the screening process for the LLM study. The healthy control (HC) group comprised of eight age-matched healthy individuals (mean age = 66.95, SD = 6.4), with MMSE scores above 28 (mean = 28.65, SD = 0.98) and MoCa scores above 27 (mean = 26.35, SD = 1.70), in accord with Greek norms (Fountoulakis et al., 2000; Konstantopoulos et al., 2016). The MCI group suffered from impairments in multiple domains, with memory impairment being predominant. As part of the LLM study, eyes closed resting-state EEG activity was recorded for 5 minutes, with a sampling frequency of 500 Hz. EEG data were recorded using a Nihon Kohden JE-207A device with 57 active EEG electrodes positioned according to the International 10–10 system. The remaining seven electrodes were: two for reference on the mastoids, a ground electrode, vertical and horizontal electro-ophthalmogram and electrocardiogram, the latter two using bipolar electrodes. All electrode impedances were kept below 2 kOhms and the quality of the signal was monitored during the whole recording.

The PPA group comprised 14 right-handed individuals (mean age = 68.57, SD = 7.9) from a treatment investigation at the Johns Hopkins University (JHU). PPA diagnosis and variant classification (9 lvPPA, 5 nfvPPA) was based on the consensus criteria published by a group of experts in 2011 (Gorno-Tempini et al., 2011). The MMSE scores for the PPA group ranged between 12 and 29 (mean = 22.14, SD = 4.93) and MoCA scores ranged between 9 and 26 (mean = 16.93, SD = 5.09). Electrophysiological data included eyes-closed resting-state 8-channel EEG recordings, lasting for 2–4 minutes. EEG data were recorded using the Neuroelectrics StarStim 8 system with the electrodes positioned according to the International 10–10 system, sampled at 500 Hz, with a maximum impedance value of 2 kOhms. The PPA group included more participants than the other groups, to account for the shorter duration of the PPA EEG recordings.

Participants from both studies gave written consent for their participation according to Helsinki declaration. The protocol for the MCI and healthy control participants recruitment was approved by the Bioethics Committee of the School of Medicine of the Aristotle University of Thessaloniki, as well as the Board of the Greek Association of Alzheimer’s Disease and Related Disorders (GAADRD). The PPA participants were recruited under the following protocols approved by the Johns Hopkins Medicine Institutional Review Board: IRB 00201027, and IRB 00229164.

### 2.2 Data pre-processing

In order to incorporate the EEG signals from two heterogeneous sources, we retained all eight channels from the PPA dataset and the same eight channels from the LLM dataset, namely channels F7, T7, CP3, P5, F8, T8, CP4, and P6. This facilitated the incorporation of these two datasets into one for the purpose of the current analyses.

Prior to feature extraction, the recorded data were pre-processed to remove content unrelated to brain activity and eliminate noise artifacts. The process involved several steps which are detailed in Chriskos et al. (2018). Briefly, the mean of each electrode was subtracted from its respective activation so that all signals have a mean value of zero, followed by the application of 5 s order Butterworth filters. The order in which the filters were applied was, first, a high pass filter at 0.5 Hz (remove the direct current, DC, component), second, a low pass filter at 100 Hz (remove high frequency content irrelevant to the EEG), and, finally, three band-stop filters centered at the powerline frequency and its first two harmonics, depending on the region in which the recordings took place (50 Hz for PPA recordings and 60 Hz for LLM and healthy recordings). The final pre-processing step was the segmentation of the EEG data into epochs of 8.192 s each. In some cases, where strong linear trends could not be removed by filtering, the least-squares fit straight line was calculated and subtracted from the data (Schlögl, 2002). Re-referencing was carried out using the common average re-referencing method (Kayser and Tenke, 2010). Independent Component Analysis (ICA) was not applied since the small number of channels in one of the datasets prohibited its implementation.

### 2.3 Feature extraction

A total of 113 features were extracted per epoch and were used for the classification analyses. These features included (i) 56 FC values across the 8 channels, (ii) 12 values based on graph theory metrics, and (iii) 45 values reflecting the energy of various rhythms. The extraction of these features is described in more detail below.

#### 2.3.1 Functional connectivity

FC features between electrode pairs were estimated using the Relative Wavelet Entropy (RWE) (Rosso et al., 2001; for more details on the method used, see Supplementary Appendix 1). The FC features used in this study represent the degree to which the energy distribution between the delta (0.5–4 Hz), theta (4–8 Hz), alpha (8–12 Hz), beta (12–20 Hz), and gamma (20–50 Hz) EEG rhythms are similar between each electrode pair.^1^ Since RWE provides a non-symmetric functional connectivity matrix (for details, see Supplementary Appendix 1), we extracted a total of 56 FC features across the 8 channels.

#### 2.3.2 Graph metrics

The RWE functional connectivity matrix (derived as described above and in Supplementary Appendix 1), also known as *synchronization* matrix, can be regarded as a graph adjacency matrix, from which graph metrics can be calculated to assess the overall connectivity between each pair of electrodes (Deuker et al., 2009; Frantzidis et al., 2014). In total, 12 values were extracted based on the following five graph theory metrics:

1. the Clustering Coefficient (CC), quantifying the strength of immediate neighbor connectivity (eight values - one value per electrode),
2. the Characteristic Path Length (CPL) the sum of the connectivity values in the shortest path connecting all nodes (one value for all electrodes),
3. the Characteristic Path Efficiency (CPE) the efficiency of the above path (one value for all electrodes),
4. the Connection Density (CD), the ratio of the number of connections present in the graph divided by the total number of possible connections (one value for all electrodes),
5. the Small World Metric (SW) is calculated by dividing the mean CC and CPL of the given graph as a ratio of the same metrics derived from a set of random graphs with the same size, which quantifies the ease of information transfer between the nodes in the graph (one value for all electrodes).

#### 2.3.3 EEG rhythm energy ratio

The final set of features reflected the rhythm energy ratios of the EEG signal. The energy ratios of the five main brain rhythms were calculated for the whole signal, as well as for each electrode separately. Specifically, we focused on the power ratios of the delta (*δ*), theta (*Θ*), alpha (*α*), beta (*β*) and gamma (*γ*) rhythms over the total energy of the signal. The energy was calculated as described in Supplementary Appendix 2 - Equation 2. In total 45 values reflecting the energy of various rhythms were extracted, five for each rhythm per electrode and another five for each rhythm, for the whole EEG.

### 2.4 Classification analyses

The classification analysis evaluated how successful different classifiers are in dissociating the three groups based on the features extracted from the EEG data. The entire set of 113 features was used for the classification analyses. Our classification analysis had two levels: epoch-level and participant-level. First, we conducted a classification analysis at the epoch-level, to assign a group label to each epoch. Then, for the participant-level classification, for each participant, the labels of the classified epochs were counted, and the participant was classified in the group with the majority of labels. The classification analysis required the separation of the dataset into a training and test sets, that were used to train and evaluate the classifiers, respectively. A ratio of approximately 70–30 (for training and testing, respectively) was used for all analyses. The number of epochs per participant group and classification set are presented in Table 1.

**Table 1:** Number of epochs per participant (Epochs) and number of participants (PP) for each of the three groups, separately for the train and test sets, as well as the total numbers.

Set	HC		MCI		PPA		Total	
	Epochs	PP	Epochs	PP	Epochs	PP	Epochs	PP
Train	270	6	225	5	210	10	705	22
Test	91	2	94	3	84	4	269	8
Total	361	8	319	8	294	14	974	30

Classification was carried out using the k-Nearest Neighbor (k-NN) and Support Vector Machines (SVM) classifiers, with k-NN mostly used for linearly separable groups, and SVM used for more complex schemes. Initially Bayesian Optimization (BO) (Snoek et al., 2012) was used in order to attain approximate good parameter value ranges and combinations. However, BO is only applied on the training set, not the testing set. Therefore, it is not expected to provide the best possible results. The values were manually optimized based on the accuracy attained by each classifier on the test set.

The main parameters optimized for the k-NN classifier are the number of nearest neighbors *k* and the distance metric used. In our experiments we used the Cityblock (Manhattan) distance, the Euclidean distance and the Cosine similarity metric. For the SVM classifiers, we used three different kernels, i.e., linear, polynomial and radial basis function (RBF). The polynomial kernel is governed by its degree parameter, while the RBF *σ* was optimized. The σ parameter adjusts the width of the RBF kernel. Other combinations of parameters were also used but provided similar or poorer results. The use of more complex classifiers (neural networks, convolutional neural networks, extreme learning machines) was prohibited by the small number of available data. Analyses were conducted for binary (i.e., two groups at a time) classification between all class pairs, namely HC vs. MCI, HC vs. PPA, PPA vs. MCI, as well as across the three groups, that is HC vs. MCI vs. PPA.

In order to assess the importance of each calculated feature we used the C4.5 algorithm (Quinlan, 2014). The C4.5 algorithm was applied with no limit on the maximum depth, merging the leaves from the same parent node that have risk values equal or greater compared to the parent, prior probabilities calculated on the training set and with pruning enabled. This algorithm is used to generate a decision tree by identifying the features that, when removed from the rest, maximize the normalized information gain ratio. For each one of these features a decision node is created in order to classify the training samples. This process is repeated until all samples belong to the same group or the remaining features do not provide information gain. Since the features are selected by order of importance, this algorithm can be used as a feature ranking method.

## 3 Results

### 3.1 Classification

As mentioned earlier, a large set of combinations of classifiers and parameters were tested. The results are presented in Table 2, which shows in bold font the row that corresponds to the classifiers with the highest classification accuracy on the test set for each classification scheme. Below we discuss the results of the test sets for the two classifiers that achieved the highest accuracy scores in each group comparison, both at the epoch- and participant-levels. As a reminder, our classification analysis had two levels: epoch-level and participant-level. First, we conducted a classification analysis at the epoch-level, to assign a group label to each epoch, and then, for the participant-level, the labels of the classified epochs were counted separately for each participant, and the participant was classified as belonging in the group with the majority of labels.

**Table 2:** Classification results both at the epoch level (Epoch accuracy) as well as the participant level (Participant accuracy), separately for each comparison of interest.

HC-MCI						
Classifier	Parameters		Epoch accuracy		Participant accuracy	
			Train	Test	Train	Test
**k-NN**	**Cityblock**	**1**	**100.00%**	**76.11%**	**100.00%**	**100.00%**
k-NN	Euclidean	3	99.54%	67.78%	100.00%	80.00%
k-NN	Cosine	3	99.77%	68.33%	100.00%	80.00%
SVM	Linear	–	99.76%	61.67%	100.00%	80.00%
SVM	Poly	d = 11	99.74%	62.22%	100.00%	80.00%
SVM	RBF	σ = 0.00001	100.00%	65.56%	100.00%	80.00%

HC-PPA						
Classifier	Parameters		Epoch accuracy		Participant accuracy	
			Train	Test	Train	Test
k-NN	Cityblock	101	98.36%	74.85%	100.00%	83.33%
k-NN	Euclidean	93	96.04%	67.43%	100.00%	66.67%
k-NN	Cosine	111	92.29%	68.00%	100.00%	66.67%
SVM	Linear	–	98.54%	58.29%	100.00%	66.67%
SVM	Poly	d = 5	93.54%	56.00%	100.00%	66.67%
**SVM**	**RBF**	**σ = 0.7**	**99.58%**	**90.29%**	**100.00%**	**100.00%**

MCI-PPA						
Classifier	Parameters		Epoch accuracy		Participant accuracy	
			Train	Test	Train	Test
k-NN	Cityblock	65	89.84%	69.94%	93.33%	71.43%
k-NN	Euclidean	77	88.77%	72.83%	100.00%	71.43%
k-NN	Cosine	63	90.91%	76.88%	100.00%	85.71%
SVM	Linear	–	98.66%	82.66%	100.00%	71.43%
SVM	Poly	d = 3	99.20%	85.55%	100.00%	85.71%
**SVM**	**RBF**	**σ** **= 1.1**	**100.00%**	**91.91%**	**100.00%**	**100.00%**

HC-MCI-PPA						
Classifier	Parameters		Epoch accuracy		Participant accuracy	
			Train	Test	Train	Test
k-NN	Cityblock	7	98.91%	54.17%	100.00%	66.67%
k-NN	Euclidean	3	99.20%	53.03%	100.00%	66.67%
k-NN	Cosine	3	99.38%	54.54%	100.00%	66.67%
SVM	Linear	–	98.91%	45.08%	100.00%	55.56%
SVM	Poly	d = 7	99.69%	46.21%	100.00%	55.56%
**SVM**	**RBF**	**σ = 1.38**	**100.00%**	**54.17%**	**100.00%**	**77.78%**

k-NN, k nearest neighbor; SVM, support vector machine; RBF, radial basis function. Bold values indicate highest accuracy rate achieved on the test set for each classification experiment.

#### 3.1.1 HC vs. MCI

When classifying between HC and MCI patients, the highest accuracy was achieved by the k-NN classifier with *k* = 1 using the Cityblock distance. For the test set, accuracy at the epoch level was at 76.11%, while at the participant level it was at 100%. The second highest accuracy rate was achieved by the k-NN with 3 nearest neighbors and the Cosine similarity metric. For the test set, accuracy at the epoch level was at 68.33%, while at the participant level it was at 80%.

#### 3.1.2 HC vs. PPA

When classifying between the HC and PPA patients, the highest accuracy was achieved by the SVM classifier with an RBF kernel with *σ* = 0.7 and the box constraint equal to 1. For the test set, accuracy at the epoch level was at 90.29% while at the participant level it was at 100%. The second highest accuracy rate was achieved by a coarse k-NN classifier with *k* = 101 and the Cityblock distance metric. For the test set, accuracy at the epoch level was at 74.85%, while at the participant level it was at 83.33%.

#### 3.1.3 MCI vs. PPA

When classifying between MCI and PPA patients, the highest accuracy was achieved by the SVM classifier, using the RBF kernel with σ = 1.1 and the box constraint equal to 10. For the test set, accuracy at the epoch level was at 91.91% while at the participant level it was at 100%. The second highest accuracy rate was achieved by the SVM classifier with a third-degree polynomial kernel with a box constraint, as in the previous case, equal to 10. For the test set, accuracy at the epoch level was at 85.55% while at the participant level it was at 85.71%.

#### 3.1.4 HC vs. MCI vs. PPA

When classifying between all three groups, that is HC, MCI and PPA patients, the highest accuracy was achieved by the SVM with a RBF kernel, a σ value of 1.38 and a box constraint value equal to 1. For the test set, accuracy at the epoch level was at 54.17% while at the participant level it was at 77.78%, misclassifying one PPA patient as MCI, and one MCI patient as healthy. The other classifiers achieved even lower classification accuracies.

For each of the three pair-wise comparisons, the highest performing classifier achieved 100% accuracy at the participant-level. In the case of the three-way classification, the highest performing classifier achieved 77.78% accuracy at the participant-level. The misclassified cases were one PPA patient classified as MCI and one MCI patient classified as HC. Confusion matrices for epoch-based classification are provided in Supplementary Appendix 2.

**Figure 1:**
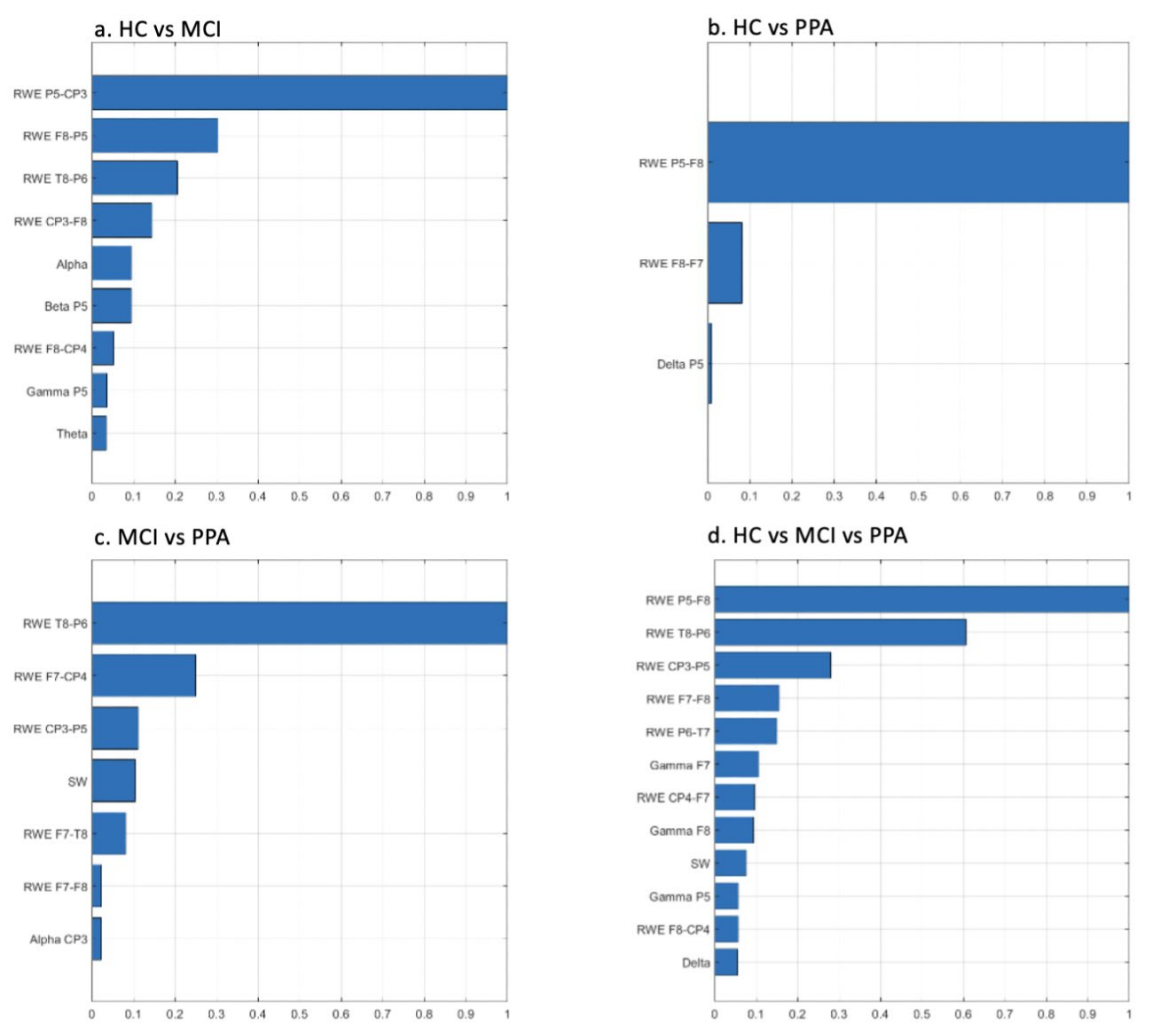
Feature ranking graphs for: **(A)** HC vs. MCI, **(B)** HC vs. PPA, **(C)** MCI vs. PPA, **(D)** HC vs. MCI vs. PPA. Rank values have been normalized to render comparisons easier. RWE, Relative Wavelet Entropy; SW, Small World Metric.

The feature ranking results for each classification approach are presented in graphical format in Figure 1. In both cases, the features are presented in decreasing rank value. The number of features in each classification scheme are different depending on the similarity of the differentiated groups.

### 3.2 EEG features per group

In this section, we provide a description for each group with respect to the various features that were used to generate the above classification results (in section 3.1): (I) FC values, (II) Graph theory metrics, and (III) Energy rhythms. It is important to note that based on the steps followed to calculate the various features, for each feature there is just one value per group (rather than per individual). Therefore, a statistical comparison of these features between groups is not possible.

#### 3.2.1 Functional connectivity values

**Figure 2:**
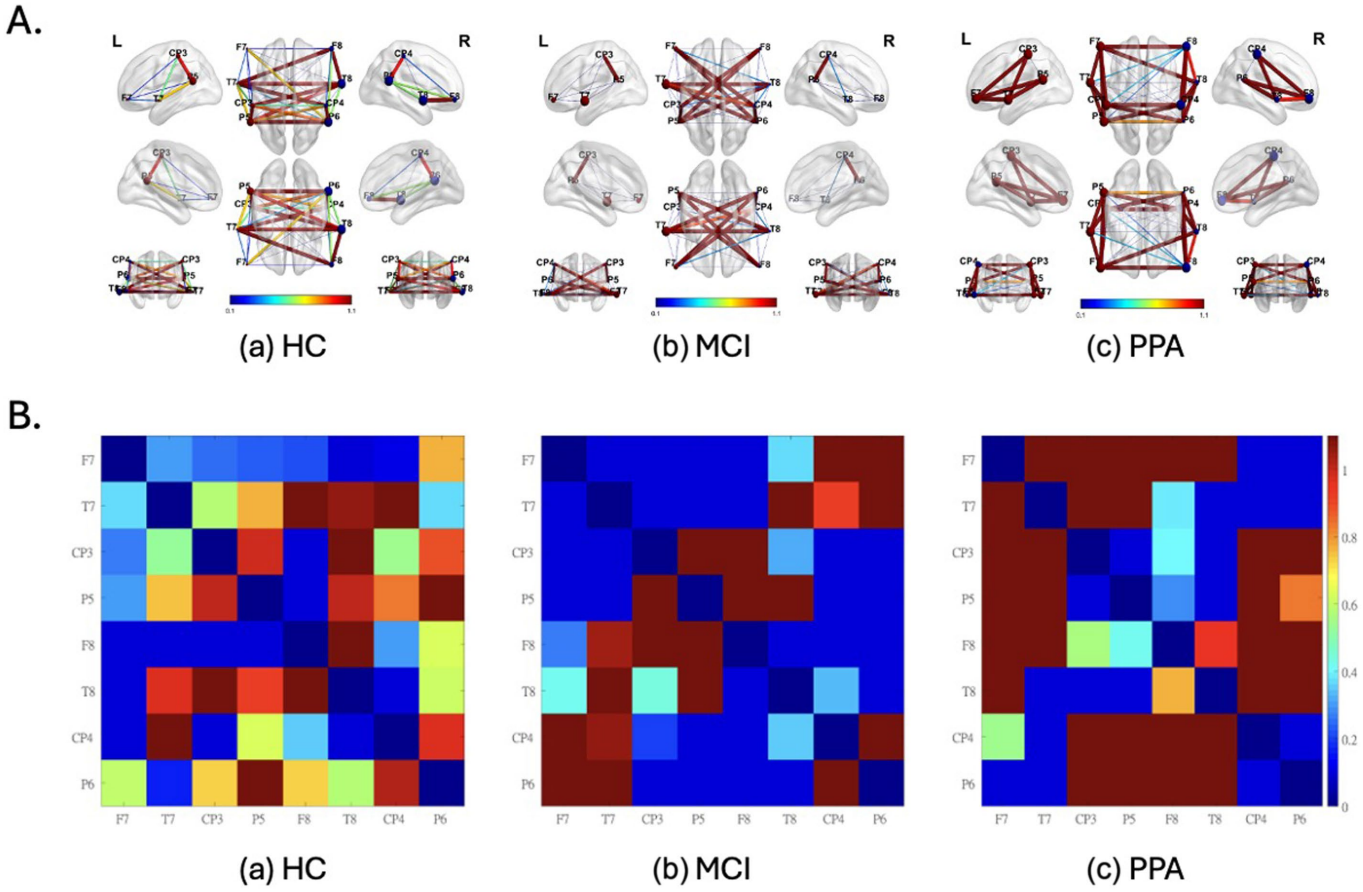
Functional Connectivity (FC) values superimposed on a brain image **(A)** and represented in a matrix format **(B)** as well, for each group (a) HC, (b) MCI and (c) pPA. **(A)** L, left hemisphere; R, right hemisphere. Warm colors (red) and thicker connections suggest higher FC values, while colder colors (blue) and thinner connections suggest lower FC values. The FC values were inverted to represent similarity values, and consecutively normalized to render the differences between the groups more apparent. Also, the nodes represent EEG electrodes. The brain surface is presented for demonstration purposes only. Diagonal values (i.e., of identical signal) are set to zero. The size of the spheres represents the node degree. **(B)** Electrodes with odd numbers represent left hemisphere locations whereas the opposite is true for electrodes with even numbers. For their approximate positions refer to **(A)**. FC values have been normalized to render the differences more apparent.

Figures 2A,B graphically show the strength of FC between each pair of electrodes. Figure 2A shows the FC values superimposed on a brain image, while in Figure 2B, the same FC values are represented in a matrix format for ease of readability. Table 3 presents the average LMC, RMC, and IMC values per group.

**Table 3:** Mean connectivity values per hemisphere and between the two hemispheres per group.

	*LMC*	*RMC*	*IMC*
HC	0.10	0.12	0.15
MCI	0.05	0.06	0.17
PPA	0.18	0.17	0.14

LMC, left mean connectivity; RMC, right mean connectivity; IMC, interhemispheric mean connectivity.

#### 3.2.2 Graph theory metrics

Table 4 provides the average values of the graph theory metrics for each group. Overall, CC is lower in PPA in all electrodes of both hemispheres studied here relatively to MCI and HC.

**Table 4:** Mean graph theory metrics per group.

	*CC-F7*	*CC-T7*	*CC-CP3*	*CC-P5*	*CC-F8*	*CC-T8*	*CC-CP4*	*CC-P6*
HC	0.626	0.631	0.63	0.636	0.629	0.626	0.631	0.634
MCI	0.635	0.657	0.652	0.67	0.648	0.664	0.652	0.673
PPA	0.581	0.617	0.616	0.62	0.594	0.617	0.608	0.617

	*CPL*	*CPE*	*CD*	*SW*
HC	1.615	0.636	0.632	1.297
MCI	1.555	0.661	0.66	1.257
PPA	1.687	0.616	0.615	1.352

CC, clustering coefficient, per electrode; CPL, characteristic path length; CPE, characteristic path efficiency; CD, connection density; SW, small world metric.

#### 3.2.3 Energy rhythms

**Figure 3:**
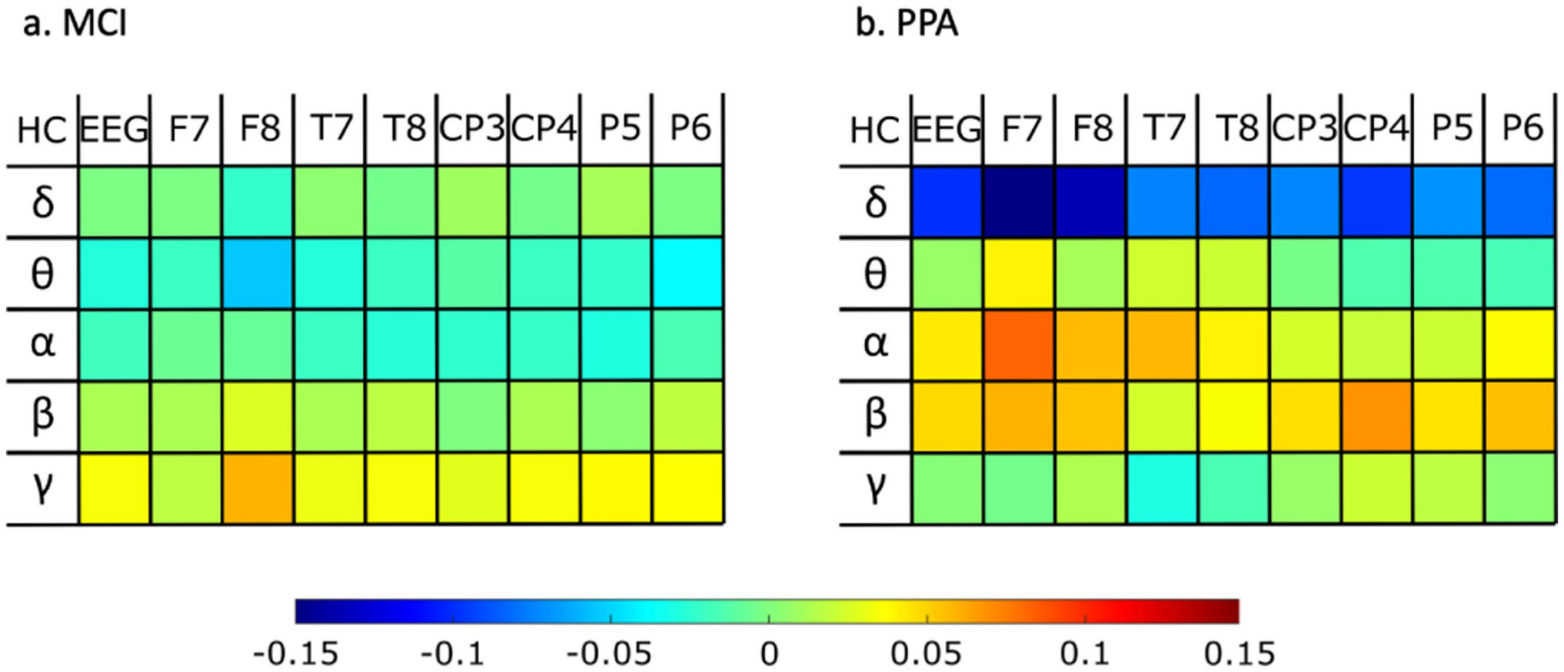
Differences of rhythm energy ratios for whole EEG and for each electrode separately, for **(A)** MCI against healthy controls, and **(B)** PPA against healthy controls. Warm colors (red) indicate a higher value for the healthy control group as opposed to the patient group, while colder colors (blue) indicate a higher value for the patient group as opposed to the healthy control group.

Figure 3 shows how the two patient groups, MCI and PPA, compare against the HC group with respect to the power of each energy rhythm at each of the eight channels, as well as averaged across all channels.

## 4 Discussion

This study investigated classification accuracy for individuals with PPA and MCI as compared to HC using low-density EEG data. Specifically, we evaluated how functional connectivity values, graph theory metrics derived from the functional connectivity graph, as well as energy rhythms can allow us to distinguishing the three groups from one another using EEG data from 8 electrodes. Providing a detailed description of group-specific profiles and of group differences is beyond the scope of the current study. However, a discussion of the differences between groups detected based on the features we used for classification in the current study is provided below. As mentioned earlier (section 3.2.), given our methodology, a statistical comparison of these features between groups is not possible. Thus, the discussed differences between groups are only indicative and future studies are needed to assess their statistical significance.

### 4.1 Classification

The results showed distinct patterns of functional connectivity and rhythm patterns across the three groups which allowed for high classification accuracies across all four comparisons of interest: (i) HC vs. MCI: 100.00%, (ii) HC vs. PPA: 100%, (iii) MCI vs. PPA: 100%, and (iv) HC vs. MCI vs. PPA: 78%. The high accuracy rates indicate that the proposed methodology is suitable for differentiating the three groups and future studies with larger sample sizes can validate its applicability.

Previous classification attempts between HC and PPA groups have been equally successful, but they involved lengthy behavioral testing (e.g., Cho et al., 2020; Cordella et al., 2017; García et al., 2022; Neophytou et al., 2019; Themistocleous et al., 2021), or expensive neuroimaging studies (e.g., Agosta et al., 2015; Bisenius et al., 2017; Bruun et al., 2019; Kim et al., 2019; Poonam et al., 2021; Spinelli et al., 2017). Classification attempts for *HC* vs. *MCI* have achieved high accuracy, such as 87% (Lagun et al., 2011), and 91% (Chriskos et al., 2021), but this is the first study that has achieved a perfect 100% accuracy. With respect to *MCI* vs. *PPA*, the one study that has compared the two groups has achieved relatively low accuracy values (59%), while in the current study we report 100% accuracy in classifying the two disorders. Finally, to the best of our knowledge, no other study has compared all three groups at the same time. While the three-way comparison shows the lowest accuracy scores, it is still considerably high (i.e., 78%).

The successful classification of the two patient groups (MCI and PPA), both in the two-way and the three-way comparisons is particularly important. In our PPA group, the majority of patients were diagnosed with lvPPA, which is primarily characterized by Alzheimer’s pathology (Gorno-Tempini et al., 2008). MCI is also often characterized by Alzheimer’s pathology (Bennett et al., 2005). Therefore, the fact that we were able to successfully classify the patients in the two groups, despite the probable overlap in the underlying pathology is especially significant.

With respect to the two classifiers used in the current study, the SVM classifiers achieved the lowest classification accuracy results for the HC vs. MCI comparison, perhaps indicating that these groups are linearly separable. While this might seem a surprising outcome, it suggests that the participant groups can be distinguished by simple classifiers, while more complex ones tend to be overtrained. On the other hand, for the HC vs. PPA and MCI vs. PPA comparisons, higher-complexity classifiers were needed to achieve high accuracy rates. These results suggest that the PPA group is not linearly separable from the other two groups, which highlights the intricate nature of this disorder and the need for further research into the neurophysiological profiles of the different variants.

Finally, we want to address any concerns that might arise for the validity of the above-mentioned classification results because of using data from heterogenous recording sources. Several measurements were taken to ensure the validity of our classification analysis and results. First, we retained the same 8 channels from both datasets, and we applied the same preprocessing pipeline in preparing the data for further analysis. To further mitigate a possible recording system effect, we used a functional synchronization metric that converts the EEG time-series into a matrix of synchronization values. Therefore, while the EEG data may be recorded differently between devices, the synchronization between the electrode pairs is mostly unaffected and not device-dependent. Another step taken to address such issues was the normalization of the data into the interval [0, 1] subtracting any direct current components that would affect the joint utilization of the initially heterogeneous sources.

### 4.2 Functional connectivity across groups

For the HC group (Figures 2Aa,Ba) we observed that the FC values cover the full spectrum of values, from 0 to 1. This was true both for within-hemisphere connections, with the strongest connection being between the posterior temporo-parietal areas (e.g., CP3-P5), as well as for the between-hemispheres connections. On the other hand, the range of values for the MCI group (Figures 2Ac,Bc) was characterized by distinctive either high or low values. Characteristic to the MCI group, are the high FC values observed between the two hemispheres, which is confirmed by the high overall interhemispheric connectivity value (IMC) for which the MCI group had the highest value out of the three groups (see Table 3).

Similar to the MCI group, the range of values for the PPA group (Figures 2Ac,Bc) was characterized by distinctive either high or low values. Characteristic to the PPA group, are the high FC values observed within each hemisphere and mostly in the left, primarily between the electrodes located above the perisylvian areas (e.g., T7-P5), and in the connections between frontal and more posterior areas of the brain (e.g., F7-T7). This is further supported by the high within-hemisphere mean connectivity values in both hemispheres (see LMC and RMC values in Table 3), for which the PPA group had the highest values out of the three groups, especially for the left hemisphere. This observation is made with the exception of the low connectivity values between the posterior temporo-parietal areas (e.g., CP3-P5) in the PPA group.

These rather different functional connectivity profiles across the three groups are, of course, a driving force for the successful classification of the three groups. Some notable differences between the groups are the particularly low left and right hemisphere mean connectivity values (LMC and RMC) of the MCI group compared to the other two groups, especially with respect to the PPA group. In other words, while the MCI group shows very *low* mean connectivity values within each hemisphere compared to HC, the PPA group shows the exact opposite pattern, that is very *high* mean connectivity values within each hemisphere compared to HC. A decline of intrahemispheric connectivity in MCI has been previously reported (Handayani et al., 2018). Increased within-hemisphere connectivity in PPA has been reported in past studies as well, both in EEG (Moral-Rubio et al., 2021) as well as fMRI resting-state functional connectivity (Tao et al., 2020). The difference between the two groups might suggest that over the course of PPA, connectivity within hemispheres is strengthened to support the various functions of each hemisphere, while in MCI, this is not the case.

### 4.3 Graph theory metrics

The graph theory metrics provided another important source for understanding how the electrophysiological profiles of the three groups are different from one another. An important graph theory metric is the clustering coefficient that characterizes each node. Clustering coefficient is an index of the functional segregation of brain networks, with high values indicating a more specialized structure of the network. In this study, the MCI group showed the highest set of values out of the three groups. The high CC values obtained in this study for the MCI group, are in line with the lower CPL value and higher CPE and CD values compared to the HC group. In other words, the high connectivity between each node’s immediate neighbors (high CC values) is associated with shorter (low CPL value) and more efficient (high CPE value) connections, and, therefore, a denser network (high CD value).

On the other hand, the PPA group showed the exact opposite pattern of results compared to the HC group. Specifically, the PPA group showed the lowest set of CC values out of the three groups, which is evidence of a less functionally segregated brain network. The low CC values obtained in this study for the PPA group are in line with the higher CPL value and lower CPE and CD values observed compared to the HC group. In other words, the low connectivity between each node’s immediate neighbors (low CC values) is associated with longer (high CPL value) and less efficient (low CPE value) connections, which lead to a less dense network (low CD value). Overall, these results suggest that the PPA group shows a less segregated network compared to the HC group. On the contrary, previous research has shown greater segregation in PPA measured with graph theory metrics in fMRI resting-state (Agosta et al., 2014; Mandelli et al., 2018; Tao et al., 2020). Interestingly, brain stimulation through transcranial Direct Current Stimulation (tDCS) improves segregation in PPA (Tao et al., 2021).

The only graph theory metric that does not show values in the expected direction given the values we get from the other graph theory metrics is SW. SW is considered an index of ease of information transfer. High CC values are expected to be associated with high SW values, because nodes that are strongly connected to their neighbors are likely to be associated with easy information transfer. In the current study, contrary to the expected pattern, SW values go in the opposite direction. Namely, the MCI group showed a lower SW value compared to the HC group (while they showed high CC values), while the PPA group showed a higher SW value compared to the HC group (while they showed low CC values).

Such a discrepancy might be caused by nodes that allow easy and efficient connectivity between otherwise far nodes. These nodes are also known as “hubs.” The low SW values for the MCI group might then be associated with the high mean connectivity between hemispheres (high IMC value), which could reflect efficient connections across faraway nodes. On the other hand, the high SW values for the PPA group might be associated with the low mean connectivity between hemispheres (low IMC value), which could reflect efficient connections only between nodes that are spatially close to each other, i.e., within the same hemisphere (see high LMC and RMC values for PPA).

### 4.4 Energy rhythms

The final set of features we investigated were the energy rhythms for each of the eight channels, as well as on average across all channels. As shown in Figure 3, the PPA group showed the greatest differences compared to the healthy control individuals. Specifically, PPA showed higher values for the delta rhythm for each of the eight channels we investigated, as well as on average across all channels. Previous work looking into changes in delta rhythm activity in MCI had also found increased delta activity in MCI as compared to HC, particularly in frontal areas (e.g., Babiloni et al., 2010; Fauzan and Amran, 2015). In the current data, while there is a trend for higher delta rhythm values in MCI vs. HC as well, the difference between these two groups did not appear to be as large as in the case of PPA vs. HC. Alpha rhythms, especially in the left frontal lobe, also showed differences between PPA and HC, but in the opposite direction. In other words, the PPA group showed lower values compared to HC. While previous research that looked into differences in alpha rhythms in MCI has also shown lower alpha values for MCI compared to HC (Jelic et al., 2000), in the current study, the MCI group did not show substantial differences compared to the HC group. Finally, previous research has reported theta rhythm slowing as a characteristic difference between PPA compared to healthy aging (Utianski et al., 2022). In the current dataset, theta rhythm in the left frontal lobe also showed a trend in the same direction.

### 4.5 Limitations

While the results if this study allow us to better understand the value of low-density EEG signal in understanding neurodegenerative disorders like PPA and MCI, there were several important limitations that need to be acknowledged. First, there was a relatively small samples size (total of 30 participants) which might limit the generalization of the results. This fact also created a challenge in correctly training the classifiers which was nonetheless achieved, addressing the concern of overfitting. Regarding the generalizability of the current findings, the small dataset size also reduces the ability of the trained classifiers to be applied to different datasets. This is particularly true for larger datasets where variability is higher. However, it is still possible, with careful optimization, to achieve high accuracy rates on datasets of various sizes.

Other limitations relate to the EEG montages. Specifically, the fact that the dataset is composed of data recorded using two different EEG montages is an important limitation of the current work. The use of different EEG montages necessarily implies variability in the internal pre-processing steps for each device, along with differences in the capabilities of the recording devices to deal with noise and other artifacts. Another issue is the unbalanced number of individuals per group due to the length of the recordings. Related to these issues, it should be noted that, in order to incorporate the EEG signals from two heterogeneous sources, we retained all eight channels from the PPA dataset and the same eight ones from the LLM dataset. Despite how challenging it was to get our groups to be as large and as balanced as possible, meaningful results were attained. Finally, an additional limitation of the low-density recordings is that the limited spatial resolution could lead to random or erroneous functional connectivity indicators, yet our results are in line with previous research that further supports the reported results.

## 5 Conclusion

Currently, distinguishing symptoms of healthy aging from those of the first stages of PPA and MCI requires lengthy cognitive and language assessments, paired with time-consuming and expensive neuroimaging data collections. The current study shows that successful classification between HC-MCI-PPA is possible with a simple 3-min EEG recording with eight electrodes. Previous attempts have been made to classify HC versus each of the two neurocognitively impaired groups separately. However, to the best of our knowledge, this is the first study that successfully classifies individuals across all three groups at the same time. The successful three-way classification renders the methodology more applicable to real-world scenarios where the distinction does not follow a strict normal vs. pathological paradigm.

Despite the methodological limitations associated with this study, these results have important implications for clinical practice since they allow for fast and accurate disease diagnosis which, in turn, allows for better management of disease progression and treatment. These results also highlight the importance of further investigating and understanding the electrophysiological changes observed in neurodegenerative diseases, particularly in PPA which is still relatively understudied. In order to gain a deeper understanding of how the three groups differ with respect to their neurophysiological profiles, future studies with larger sample sizes are needed to further compare these measurements between groups. Such studies can also allow us to establish the generalizability of the current findings with larger sample sizes and the efficient use of this methodology in a clinical setting.

## Data Availability

The datasets for this article are not publicly available due to concerns regarding participant anonymity. Data requests will be considered on an individual basis, and should be sent to: tsapkini@jhmi.edu.

## Footnotes

^1^Although frequencies above 50 Hz (i.e., high-gamma activity) can be highly relevant for the populations studied in the present paper, we excluded them because it is often argued that they are not highly reliable when measured on the skull rather than the cortical surface (Crone, 1998; Riès et al., 2017).
