# Supplementary material for: The use of low-density EEG for the classification of PPA and MCI
Source: Front Hum Neurosci. 2025 Feb 7;19:1526554. doi: 10.3389/fnhum.2025.1526554 (PMC11842309; doi:10.3389/fnhum.2025.1526554)
Supplement: Supplementary file 1 [file Data_Sheet_1.docx]

#### **Appendix A**

**Relative Wavelet Entropy (RWE) for Functional Connectivity**

The method used to extract functional connectivity features is based on the orthogonal discrete wavelet transform (Heil & Walnut, 1989), itself an extension of the discrete wavelet transform, using an orthogonal basis defined by wavelets to decompose the signal. For a uniformly sampled signal **x** decomposed into *N* levels, the orthogonal wavelet transform is calculated as:

$$X_{t}=\sum_{j=-N}^{-1} \sum_{k} C_{j}(k)\psi_{j,k}(t)$$

(1)

where *Cj(k)* are the wavelet coefficients at times *k* and ψ is a family of wavelet functions (Morlet wavelets (Cohen, 2019) were used in this study) for different combinations of *j*,*k*∈*Z*. The amplitude of the coefficients quantifies the similarity between the mother wavelet and signal **x**, in this case the EEG, while the polarity describes the sign of the similarity. The overall energy of the signal is calculated through coefficients as:

$$E_{tot}=\sum_{j<0} \sum_{k} {|C_{j}(k)|}^{2}$$

(2)

For each level of decomposition, the relative energies are calculated as the ratio of the energy of level *j* over the total energy of the signal. The relative entropy between the energies *p* and *q* of two signals, in this case for each electrode pair, is derived through the Shannon entropy (Maturana-Candelas et al., 2019) as:

$$H(p|q)=\sum_{j<0} p_{j}ln\left( \frac{p_{j}}{q_{j}} \right)$$

(3)

This allows the population of a square (same number of columns and lines) non-symmetric (the value of the pair (*x*,*y*) matrix to be different compared to (*y*,*x*)) synchronization matrix with a size equal to the number of electrodes in the signal, in this case eight. This matrix can also be regarded as a weighted directional graph from which global connectivity metrics can be extracted, which are presented below. It must be noted that the values of the main diagonal of this matrix are not retained since they contain information as to how similar each signal is with itself.

In addition to the FC features between sets of channels, we also calculated the mean functional connectivity value per hemisphere (Left Mean Connectivity - LMC; Right Mean Connectivity - RMC.), as well the mean functional connectivity between hemispheres (Interhemispheric Mean Connectivity - IMC.).

The RWE functional connectivity information was used to construct a series of graphs for each group of participants. This was achieved by using the BrainNet Viewer Matlab toolbox (Xia et al., 2013). The relevant graphs are presented in the Results section. The edges are represented by line of varying width dependent on the strength of interaction between each pair of nodes (i.e., electrodes), while the size of the nodes is scaled based on the node degree of each node (for an example, see Figure 1).

#### **Appendix B**

Confusion matrices for epoch-based classification on the test set for each pair-wise comparison as well as for the comparison across all groups.

HC-MCI

|  | HC | MCI | Precision |
| --- | --- | --- | --- |
| HC | 68 | 21 | 76.41% |
| MCI | 23 | 73 | 76.04% |
| Recall | 74.73% | 77.66% | Acc.: 76.11% |

HC-PPA

|  | HC | PPA | Precision |
| --- | --- | --- | --- |
| HC | 84 | 10 | 89.36% |
| PPA | 7 | 74 | 91.35% |
| Recall | 92.31% | 88.10% | Acc.: 90.29% |

MCI-PPA

|  | MCI | PPA | Precision |
| --- | --- | --- | --- |
| MCI | 81 | 1 | 98.78% |
| PPA | 13 | 83 | 86.46% |
| Recall | 86.17% | 98.81% | Acc.: 91.91% |

HC-MCI-PPA

|  | HC | MCI | PPA | Precision |
| --- | --- | --- | --- | --- |
| HC | 52 | 16 | 10 | 66.67% |
| MCI | 23 | 45 | 30 | 45.92% |
| PPA | 16 | 33 | 44 | 47.31 |
